# Knockdown of *DNMT1* Induces *SLCO3A1* to Promote Follicular Growth by Enhancing the Proliferation of Granulosa Cells in *Mammals*

**DOI:** 10.3390/ijms25052468

**Published:** 2024-02-20

**Authors:** Shuo Li, Liqing Zeng, Fen Miao, Nian Li, Weili Liao, Xiaofeng Zhou, Yongcai Chen, Hongyan Quan, Yingting He, Hao Zhang, Jiaqi Li, Xiaolong Yuan

**Affiliations:** Guangdong Laboratory of Lingnan Modern Agriculture, National Engineering Research Center for Breeding Swine Industry, State Key Laboratory of Swine and Poultry Breeding Industry, Guangdong Provincial Key Laboratory of Agro-Animal Genomics and Molecular Breeding, College of Animal Science, South China Agricultural University, Guangzhou 510642, China; ls06414000@163.com (S.L.); zengliqing0302@163.com (L.Z.); miaofen0329@163.com (F.M.); linian1021@126.com (N.L.); laoveli1028@163.com (W.L.); zxf@scau.edu.cn (X.Z.); cyc0915@stu.scau.edu.cn (Y.C.); quanhongyan2022@163.com (H.Q.); he_yingting@163.com (Y.H.); zhanghao@scau.edu.cn (H.Z.)

**Keywords:** *DNMT1*, *SLCO3A1*, proliferation, follicular growth

## Abstract

In female mammals, the proliferation and apoptosis of granulosa cells (GCs) have been shown to determine the fate of follicles. DNA methyltransferases (DNMTs) and *SLCO3A1* have been reported to be involved in the survival of GCs and follicular growth. However, the molecular mechanisms enabling DNMTs to regulate the expression of *SLCO3A1* to participate in follicular growth are unclear. In this study, we found that the knockdown of *DNMT1* enhanced the mRNA and protein levels of *SLCO3A1* by regulating the chromatin accessibility probably. Moreover, *SLCO3A1* upregulated the mRNA and protein levels of *MCL1*, *PCNA,* and *STAR* to promote the proliferation of GCs and facilitated cell cycle progression by increasing the mRNA and protein levels of *CCNE1*, *CDK2,* and *CCND1*, but it decreased apoptosis by downregulating the mRNA and protein levels of *CASP3* and *CASP8*. Moreover, *SLCO3A1* promoted the growth of porcine follicles and development of mice follicles. In conclusion, the knockdown of *DNMT1* upregulated the mRNA and protein levels of *SLCO3A1*, thereby promoting the proliferation of GCs to facilitate the growth and development of ovarian follicles, and these results provide new insights into investigations of female reproductive diseases.

## 1. Introduction

As the basic functional unit of ovaries [1], the normal growth, development and ovulation of follicles are necessary for the generations of mammals [2]. In *humans*, a series of female reproductive diseases such as polycystic ovary syndrome (PCOS) [3,4], endometriosis [5], and dysfunction of ovarian reserve [6] present the clinical feature of abnormal follicular growth. However, no effective therapy treats and cures these devastating diseases. Follicular growth is a complex multicellular process, and the granulosa cells (GCs) have been shown to be the main supporting and regulating cells in follicles [7,8]. Previous studies have proved that the proliferation of GCs ensures the follicular development and ovulation [2,9,10], while the excessive apoptosis of GCs leads to follicular atresia [7,11,12]. Numerous studies have indicated that the DNA methylation catalyzed by DNA methyltransferases (DNMTs) regulates the transcription of genes via altering the chromatin structure to be involved in follicular growth [13]. DNA hypermethylation is often thought to be associated with gene silencing, whereas DNA demethylation is associated with gene activation [14,15]. Interestingly, previous studies have found that DNA methylation plays an important role in follicular growth [16,17]. The knockdown of *DNMT1* increases the expression of *RSPO2*, thereby inducing follicular growth [18]. The knockdown of *DNMTs* inhibits the methylation status of *H19*/*Igf2* to decrease the apoptosis of ovarian cells [19]. *DNMT1* prevents follicular growth by mediating lncRNA *IFFD* inhibition proliferation and promoting the apoptosis of GCs [20]. These results suggest that DNMTs may regulate GCs function to modulate follicular growth by directly targeting genes, but the molecular mechanism behind this process is still not fully understood.

The solute carrier organic anion transporter family member 3A1 (*SLCO3A1*) is one of the uptake transporters that belongs to the solute carrier family [21]. Research revealed that a lower expression of *SLCO3A1* inhibits the transmembrane transport of E1S to decrease the proliferation of breast cancer cells [22]. Notably, the *SLCO3A1* has been demonstrated to regulate the proliferation and apoptosis of ovarian GCs in rats with PCOS [23], indicating the potential function of *SLCO3A1* in follicular growth. Unfortunately, the specific mechanisms by which *SLCO3A1* is medicated by DNMTs involve the follicular growth via regulating the proliferation and apoptosis of GCs, which remains to be further explored.

Hence, we aimed to investigate how DNMTs regulate the expression of *SLCO3A1* to modulate the biological function of GCs and the growth of follicles. In this study, we found that the knockdown of *DNMT1* could regulate chromatin accessibility, increase the level of *SLCO3A1* to inhibit the apoptosis of GCs, and it could also induce proliferation by promoting the cycle progression of GCs. Moreover, *SLCO3A1* facilitated E2 secretion to regulate follicular growth. These results might provide a therapeutic strategy for treating female reproductive disorders.

## 2. Results

### 2.1. SLCO3A1 Promotes the Proliferation of GCs

**Figure 1:**
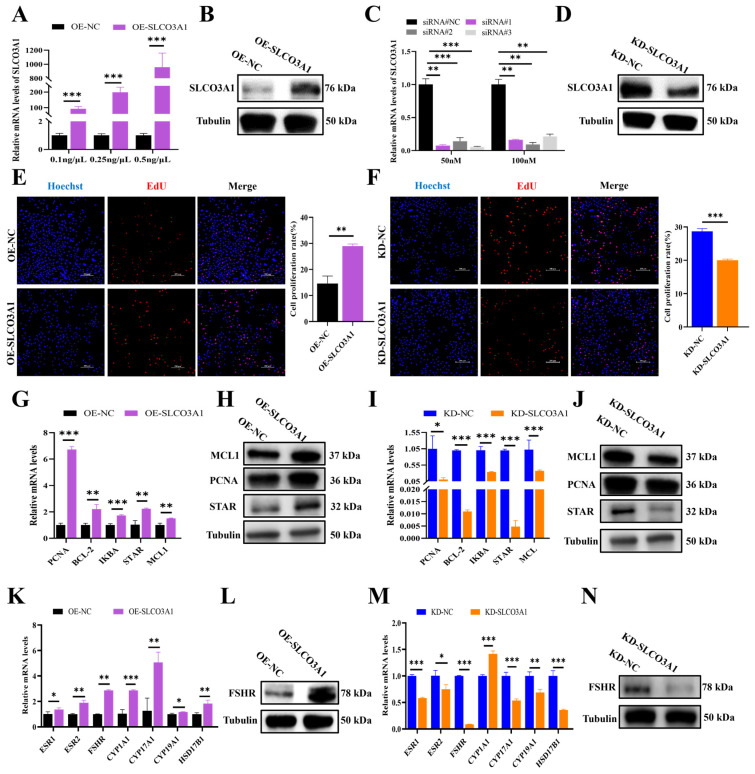
*SLCO3A1* promotes the proliferation of GCs. (**A**) RT-qPCR analysis of *SLCO3A1* expression in GCs transfected with different concentrations of pcDNA3.1 (OE-NC) or pcDNA3.1-SLCO3A1 (OE-SLCO3A1). (**B**) Western blot analysis of SLCO3A1 transfected with 0.5 ng/µL of OE-NC or OE-SLCO3A1. (**C**) RT-qPCR analysis of *SLCO3A1* expression in GCs transfected with 50 nM or 100 nM of siRNAs (siRNA#NC, siRNA#1, siRNA#2, siRNA#3). (**D**) Western blot analysis of SLCO3A1 transfected with 50 nM of siRNA#3. EdU detection of proliferation rate transfected with OE-SLCO3A1 (**E**) or KD-SLCO3A1 (**F**). Scale bar = 200 μm. RT-qPCR analysis of proliferation-related genes transfected with OE-SLCO3A1 (**G**) or KD-SLCO3A1 (**I**). Western blot analysis of MCL-1, PCNA and STAR transfected with OE-SLCO3A1 (**H**) or KD-SLCO3A1 (**J**). RT-qPCR analysis of estrogen pathway-related genes transfected with OE-SLCO3A1 (**K**) and KD-SLCO3A1 (**M**). Western blot analysis of FSHR transfected with OE-SLCO3A1 (**L**) and KD-SLCO3A1 (**N**). All of the above experimental results were obtained in the COV434 cells. * *p* < 0.05, ** *p* < 0.01, and *** *p* < 0.001.

To investigate the impact of *SLCO3A1* on the proliferation of GCs, empty plasmids (named OE-NC), overexpression plasmids of *SLCO3A1* (named OE-SLCO3A1), small interfering RNA (siRNA) of NC (named KD-NC) and siRNAs of *SLCO3A1* (named KD-SLCO3A1) were transfected into COV434 cells. The mRNA and protein levels of *SLCO3A1* were significantly upregulated by OE-SLCO3A1 treatment compared to OE-NC (Figure 1A,B), and 0.5 ng/μL of OE-SLCO3A1 was chosen for subsequent experiments. We found that the mRNA and protein levels of *SLCO3A1* were significantly reduced by KD-SLCO3A1 (siRNA#1, siRNA#2, siRNA#3) treatment compared to KD-NC (Figure 1C,D) in the GCs. And we used 50 nM of siRNA#3 for subsequent experiments. The 5-Ethynyl-2′-deoxyuridine (EdU) results showed that the proliferation rate of GCs was significantly increased by OE-SLCO3A1 treatment compared to OE-NC (Figure 1E), but it was significantly decreased by KD-SLCO3A1 treatment compared to KD-NC (Figure 1F). The mRNA and protein levels of proliferation-related genes (e.g., *MCL1*, *PCNA*, and *STAR*) were increased by OE-SLCO3A1 treatment compared to OE-NC. (Figure 1G,H). Meanwhile, the mRNA and protein levels of *MCL1*, *PCNA*, and *STAR* were decreased by KD-SLCO3A1 treatment compared to KD-NC (Figure 1I,J). Moreover, the mRNA and protein levels of *FSHR* were significantly increased by OE-SLCO3A1 treatment compared to OE-NC (Figure 1K,L), while the opposite result was found by KD-SLCO3A1 treatment (Figure 1M,N). These results suggested that *SLCO3A1* promoted the proliferation of GCs.

### 2.2. SLCO3A1 Promotes the Cell Cycle-Related Processes but Inhibits the Apoptosis of GCs

**Figure 2:**
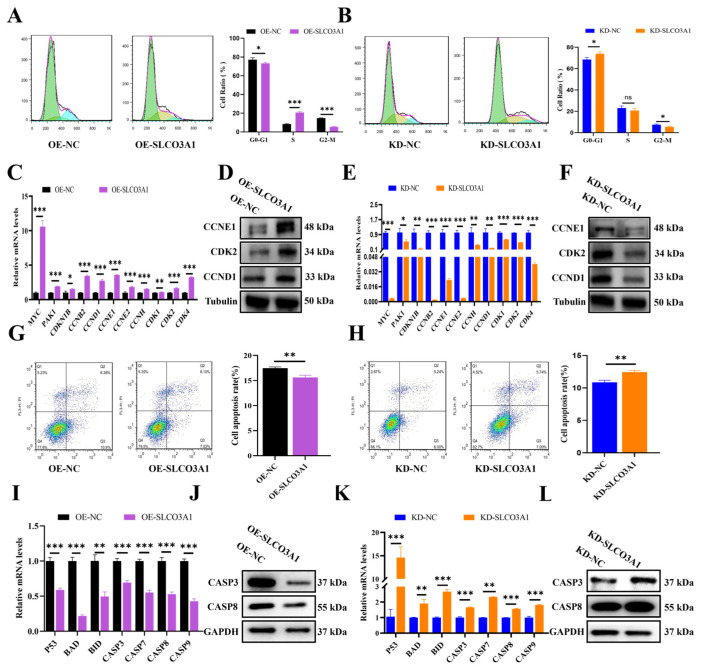
*SLCO3A1* promotes the cell cycle-related processes but inhibits the apoptosis of GCs. The cell cycle distribution was analyzed by flow cytometric transfected with OE-SLCO3A1 (**A**) or KD-SLCO3A1 (**B**). Green: cells in the G0-G1 phase, Yellow: cells in S phase, Blue: cells in G2-M phase. (**C**) RT-qPCR analysis of cycle-related genes expression transfected with OE-NC or OE-SLCO3A1. (**D**) Western blot analysis of CCNE1, CDK2 and CCND1 transfected with OE-NC or OE-SLCO3A1. (**E**) RT-qPCR analysis of cycle-related genes expression transfected with KD-NC or KD-SLCO3A1. (**F**) Western blot analysis of CCNE1, CDK2 and CCND1 transfected with KD-NC or KD-SLCO3A1. Flow cytometry assay of the apoptosis rate transfected with OE-SLCO3A1 (**G**) or KD-SLCO3A1 (**H**). Q1: dead cells and cell debris, Q2: late apoptotic cells, Q3: early apoptotic cells, Q4: negative cells. RT-qPCR analysis of pro-apoptosis related genes expression transfected with OE-SLCO3A1 (**I**) or KD-SLCO3A1 (**K**). Western blot analysis of CASP3 and CASP8 transfected with OE-SLCO3A1 (**J**) or KD-SLCO3A1 (**L**). All of the above experimental results were obtained in the COV434 cells. * *p* < 0.05, ** *p* < 0.01, *** *p* < 0.001, ^ns^ *p* > 0.05.

**Figure 3:**
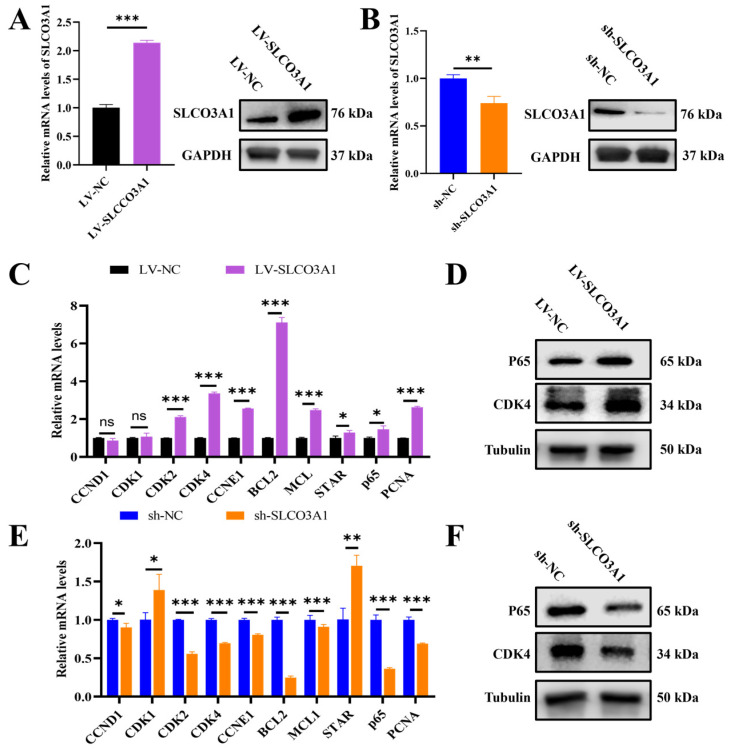
*SLCO3A1* promotes the growth of porcine follicles. The mRNA and protein levels of *SLCO3A1* of porcine follicles treated with LV-SLCO3A1 (**A**) or KD-SLOC3A1 (**B**). RT-qPCR analysis of proliferation- and cycle-related genes expression treated with LV-SLCO3A1 (**C**) or sh-SLCO3A1 (**E**). Western blot analysis of P65 and CDK4 treated with LV-SLCO3A1 (**D**) or sh-SLCO3A1 (**F**). * *p* < 0.05, ** *p* < 0.01, *** *p* < 0.001, ^ns^ *p* > 0.05.

We next investigated the effects of *SLCO3A1* on the cell cycle distribution and apoptosis of GCs. Flow cytometry results showed that the rate of G0-G1 phase cells was decreased by OE-SLCO3A1 treatment compared to OE-NC (Figure 2A), but it was increased by KD-SLCO3A1 treatment compared to KD-NC (Figure 2B) in the COV434 cells. The mRNA and protein levels of maker genes involved in the transition from G1 phase to S phase (e.g., *CCNE1*, *CDK2*, and *CCND1*) were increased by OE-SLCO3A1 treatment compared to OE-NC (Figure 3C,D), but the opposite results were found for KD-SLCO3A1 treatment (Figure 2E,F). The apoptosis rate of GCs was significantly decreased by OE-SLCO3A1 treatment compared to OE-NC (Figure 2G), while it was significantly increased by KD-SLCO3A1 treatment compared to KD-NC (Figure 2H). Furthermore, the mRNA and protein levels of pro-apoptotic genes (e.g., *CASP3* and *CASP8*) were decreased by OE-SLCO3A1 treatment compared to OE-NC (Figure 2I,J). The mRNA and protein levels of *CASP3* and *CASP8* increased by KD-SLCO3A1 treatment compared to KD-NC (Figure 2K,L). Thus, it was concluded that *SLCO3A1* facilitated the cell cycle-related processes and suppressed the apoptosis of GCs.

### 2.3. SLCO3A1 Promotes the Growth of Porcine Follicles

To further investigate the function of *SLCO3A1* in follicular growth, the ovarian follicles of pigs were subjected to lentiviral-mediated treatments including control for overexpression (LV-NC), overexpression of *SLCO3A1* (LV-SLCO3A1), control for knockdown (sh-NC), and knockdown of *SLCO3A1* (sh-SLCO3A1). It was found that the mRNA and protein levels of *SLCO3A1* were increased by LV-SLCO3A1 treatment compared to LV-NC (Figure 3A), but they were decreased by sh-SLCO3A1 treatment compared to sh-NC (Figure 3B). The mRNA and protein levels of *CDK4* and *P65* were significantly upregulated by LV-SLCO3A1 treatment compared to LV-NC (Figure 3C,D), while the opposite results were observed by sh-SLCO3A1 treatment (Figure 3E,F). Together, these data indicated that *SLCO3A1* promoted follicular growth by promoting the proliferation of GCs.

### 2.4. SLCO3A1 Promotes the Follicular Growth in Mice

**Figure 4:**
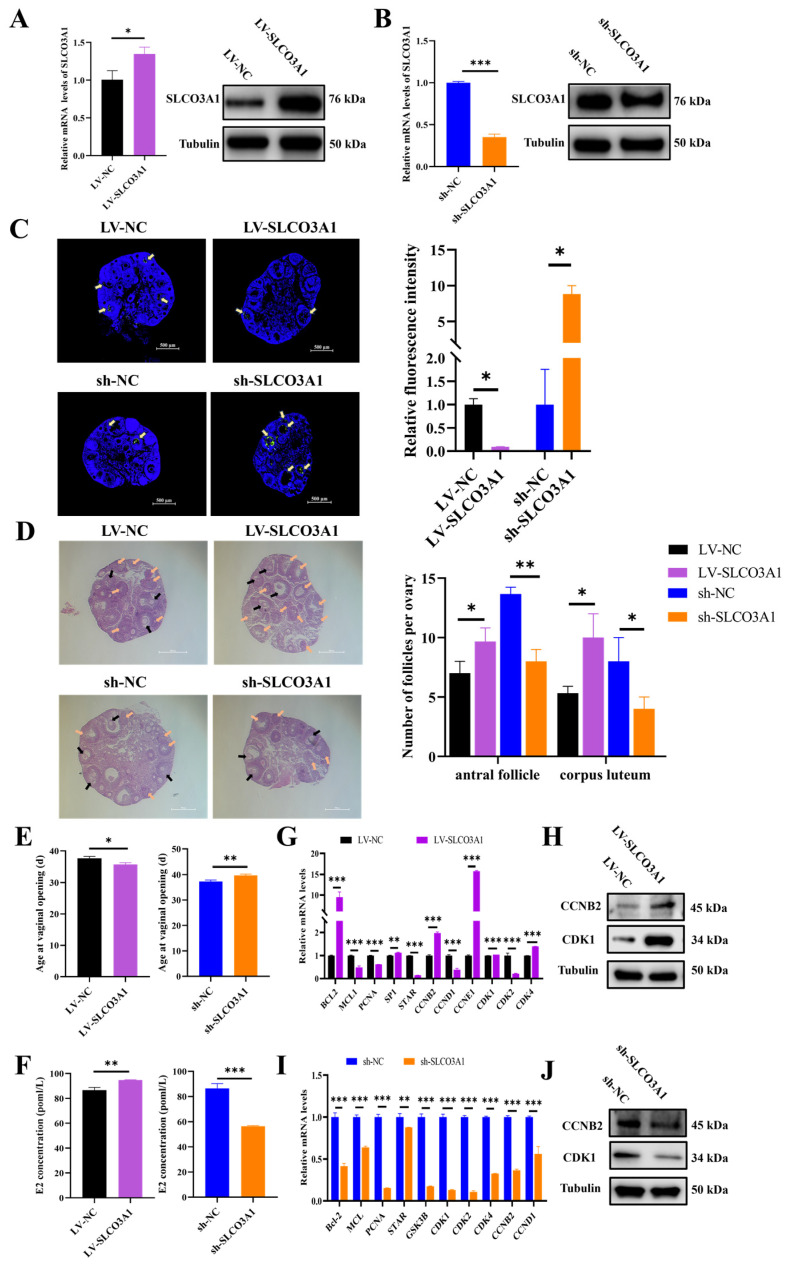
*SLCO3A1* promotes follicular growth in mice. The mRNA and protein levels of *SLCO3A1* in the ovaries of mice treated with LV-SLCO3A1 (**A**) and sh-SLCO3A1 (**B**). (**C**) The TUNEL staining was performed in mice ovaries treated with LV-SLCO3A1 or sh-SLCO3A1, and the relative fluorescence intensity was corrected by that of the control groups. Yellow arrows indicated apoptotic GCs. Scale bar = 500 μm. (**D**) The HE staining and follicular statistics of mice after their ovaries were treated with LV-SLCO3A1 or sh-SLCO3A1. Black arrows indicated antral follicles, and orange arrows indicated corpus luteum. Scale bar = 500 μm. (**E**) The age of mice at vaginal opening treated with LV-SLCO3A1 or sh-SLCO3A1. (**F**) The concentration of E2 treated with LV-SLCO3A1 or sh-SLCO3A1 was assessed by ELISA. RT-qPCR analysis of proliferation- and cycle-related genes expression treated with LV-SLCO3A1 (**G**) or sh-SLCO3A1 (**I**). Western blot analysis of CCNB2 and CDK1 treated with LV-SLCO3A1 (**H**) or sh-SLCO3A1 (**J**). * *p* < 0.05, ** *p* < 0.01, *** *p* < 0.001.

To further validate the role of *SLCO3A1* in ovarian follicular growth, LV-NC, LV-SLCO3A1, sh-NC, or sh-SLCO3A1 were injected into the mice. Compared to LV-NC, the mRNA and protein levels of *SLCO3A1* were elevated by LV-SLCO3A1 treatment (Figure 4A), while they decreased by sh-SLCO3A1 (Figure 4B). The terminal deoxynucleotidyl transferase mediated dUTP nick-end labeling (TUNEL) showed lower green fluorescence intensity with LV-SLCO3A1 treatment compared to LV-NC, but the fluorescence was higher with sh-SLCO3A1 treatment (Figure 4C). The hematoxylin and eosin (HE) staining showed that the number of antral follicles and corpus luteum were increased by LV-SLCO3A1 treatment compared to LV-NC, while this number was decreased by sh-SLCO3A1 treatment compared to sh-NC (Figure 4D). A previous study shows that the vaginal opening indicates that mice are in the estrus stage [24], and the age of mice at vaginal opening was earlier by LV-SLCO3A1 treatment compared to LV-NC, but it was significantly delayed by sh-SLCO3A1 treatment compared to sh-NC (Figure 4E). The E2 level was significantly elevated by LV-SLCO3A1 treatment compared to LV-NC, while it was significant inhibited by sh-SLCO3A1 treatment compared to sh-NC in serum (Figure 4F). The mRNA and protein levels of *CCNB2* and *CDK1* were increased by LV-SLCO3A1 treatment compared to LV-NC (Figure 4G,H), while they declined by sh-SLCO3A1 treatment (Figure 4I,J). These results suggested that *SLCO3A1* promoted the follicular maturation and eventually facilitated the ovulation and luteinization by inhibiting the apoptosis of GCs and promoting the secretion of E2 in mice.

### 2.5. Knockdown of DNMT1 Upregulates the mRNA and Protein Levels of SLCO3A1

**Figure 5:**
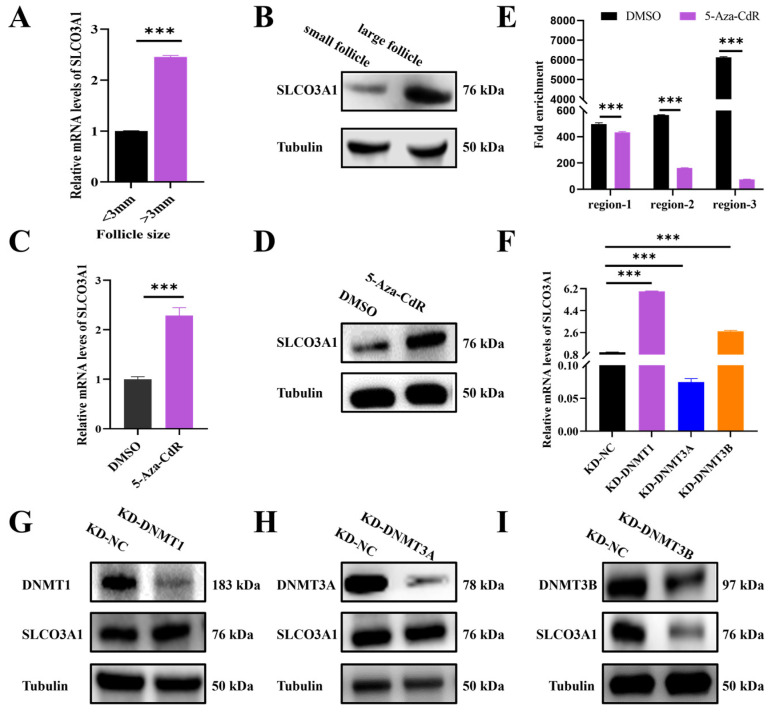
Knockdown of *DNMT1* upregulates the mRNA and protein levels of *SLCO3A1*. (**A**) RT-qPCR analysis of *SLCO3A1* expression in the small (<3 mm in diameter) and large (>3 mm in diameter) follicles. (**B**) Western blot analysis of SLCO3A1 in the small and large follicles. (**C**) RT-qPCR analysis of *SLCO3A1* expression in the DMSO or 5-Aza-CdR treated-cells. (**D**) Western blot analysis of SLCO3A1 in the DMSO or 5-Aza-CdR treated-cells. (**E**) Chromatin accessibility assay of region-1, region-2, and region-3 in the *SLCO3A1* promoter treated with DMSO or 5-Aza-CdR (region-1, +971/+1252 bp, region-2, +1433/+1557 bp, and region-3, +1623/+1831 bp, transcription start site = +2000 bp). (**F**) RT-qPCR analysis of *SLCO3A1* expression in GCs transfected with siRNAs of *DNMT1*, *DNMT3A*, or *DNMT3B*. Western blot analysis of SLCO3A1, DNMT1 (**G**), DNMT3A (**H**), and DNMT3B (**I**) in the siRNAs of *DNMT1*, *DNMT3A*, or *DNMT3B* treated-cells. *** *p* < 0.001.

To investigate the regulation of *SLCO3A1* by DNMTs in follicular growth, the expression of *SLCO3A1* in the small (<3 mm in diameter) and large (>3 mm in diameter) follicles of pigs was detected. We found that the mRNA and protein levels of *SLCO3A1* in the large follicles were significantly higher than that in the small follicles (Figure 5A,B). Moreover, the mRNA and protein levels of *SLCO3A1* were increased by 5-Aza-CdR treatment compared to DMSO (Figure 5C,D). We found that the chromatin accessibilities of region-1, region-2, and region-3 were significantly reduced in the 5-Aza-CdR treated-cells (Figure 5E). The siRNAs of *DNMT1*, *DNMT3A*, or *DNMT3B* (named KD-DNMT1, KD-DNMT3A, and KD-DNMT3B, respectively) were transfected into GCs. Both mRNA and protein levels of *SLCO3A1* displayed insignificant changes by KD-DNMT3A or KD-DNMT3B treatment (Figure 5F,H,I). But the mRNA and protein levels of *SLCO3A1* increased by KD-DNMT1 treatment (Figure 5F,G). These results demonstrated that the knockdown of *DNMT1* likely enhanced the mRNA and protein levels of *SLCO3A1* by regulating the chromatin accessibility.

## 3. Discussion

In recent years, the incidence of female reproductive disorders, such as PCOS, endometriosis, and diminished ovarian reserve, have been increasing, which is a serious threat to women’s health and fertility [25]. Follicular growth can be delayed due to a decreased proliferation of GCs and increased apoptosis of GCs, which are thought to play essential roles in the pathogenesis of female reproductive diseases [5,26,27]. However, there is no effective therapy for these disorders, and the specific mechanisms regulating follicular growth remain to be further explored. In the present study, we confirmed that the knockdown of *DNMT1* upregulated the level of *SLCO3A1*, resulted in an enhanced proliferation of GCs, increased E2 secretion, and inhibited the apoptosis of GCs, ultimately promoting follicular growth. Targeting *SLCO3A1* may be a new strategy for the clinical management of female reproductive disorders.

To explore the biological functions of *SLCO3A1* on the survival of GCs, the overexpression (Figure 1A,B) or knockdown (Figure 1C,D) of *SLCO3A1* was achieved in GCs. Accumulating studies have shown that the proliferation of GCs is an essential condition that must be present to ensure proper follicular growth [28,29,30]. The findings in the present study suggested that *SLCO3A1* promoted GCs proliferation by upregulating *PCNA*, *MCL1*, and *STAR* at the mRNA and protein levels (Figure 1E–J). *PCNA* promotes the proliferation of GCs and improves follicular growth in PCOS rats [31]. In general, cell proliferation and the cell cycle are closely connected [32]. *SLCO3A1* upregulated the expression of *CCND1*, *CCNE1*, and *CDK2*, which indicated that *SLCO3A1* could promote proliferation by facilitating cell cycle progression (Figure 2A–F). *CCND1* and *CCNE1* are crucial regulators of the cell cycle, controlling the G1/S transition [33], which could promote the proliferation of GCs [18]. Excessive apoptosis of GCs contributes to follicular atresia [34]. Our results indicated that *SLCO3A1* inhibited apoptosis by downregulating the mRNA and protein levels of *CASP3* and *CASP8* (Figure 2G–L). *CASP3* and *CASP8* are known pro-apoptotic genes that have been reported to promote the apoptosis of GCs in patients with PCOS [35,36]. We found that the expression of *SLCO3A1* in the large follicles was significantly higher than that in the small follicles (Figure 5A,B). These suggested that *SLCO3A1* might play a pivotal role as a promoting gene during follicular growth.

To further investigate the function of *SLCO3A1*, we successfully achieved the overexpression (Figure 3A) or knockdown (Figure 3B) of *SLCO3A1* by lentiviral infection using porcine follicles as a model. Our results indicated that *SLCO3A1* promoted proliferation by upregulating the mRNA and protein levels of *P65* and *CDK4* (Figure 3C–F). *P65* and *CDK4* were reported to promote the cell proliferation of GCs [37].

Referring to previous research methods [20,38], we altered the expression of *SLCO3A1* by an intraperitoneal injection of lentivirus in the ovaries of mice (Figure 4A,B). Our findings indicated that *SLCO3A1* upregulated the expression of *ESR1*, *ESR2* and *FSHR* (Figure 1K–N) as well as promoted E2 secretion (Figure 4F). Moreover, we found that *SLCO3A1* inhibited the apoptosis of GCs (Figure 4C), promoted the formation of antral follicles and corpora lutea (Figure 4D), and accelerated vaginal opening in mice (Figure 4E). Previous studies have demonstrated that *SLCO3A1* mediates the transport of steroid hormones [39]. Based on these results, we speculated that *SLCO3A1* regulated follicular growth through the transport of estrogen. Additionally, *SLCO3A1* could promote the proliferation of GCs by upregulating *CCNB2* and *CDK1* at the mRNA and protein levels (Figure 4G–J). Our study has several limitations that should be considered. Firstly, the use of five mice per treatment group in the current study may limit the generalizability of our findings. Increasing the number of mice in future studies could enhance the robustness of conclusions. Secondly, the intraperitoneal injection of LV-SLCO3A1 and sh-SLCO3A1 might impact the function of other organs, which could influence the observed changes in the mouse ovaries. Future research could use the tissue-specific gene editing mouse models to more precisely investigate the role of *SLCO3A1* in reproductive disorders. Thirdly, due to limitations in our experimental materials and methods, the number of atretic follicles was not quantified. It is better to count the number of atretic follicles to describe follicular growth.

DNMTs have been reported as a key regulator of follicular growth [40,41,42]. We found that the expression of *SLCO3A1* was significantly increased in the GCs treated with 5-Aza-CdR (Figure 5C,D) [43,44,45]. But after 5-Aza-CdR treatment, a significant reduction in chromatin accessibility was observed in the promoter region of *SLCO3A1* (Figure 5E), suggesting that changes of DNA methylation might be not in line with the changes of chromatin accessibility, and this appearance was consistent with previous findings [46]. This indicated that the process of modifying chromatin status through DNA demethylation for epigenetic regulation is intricate and merits further investigation. Moreover, our findings suggested that the mRNA and protein levels of *SLCO3A1* were upregulated by KD-DNMT1 treatment (Figure 5G). This was in line with previous studies indicating that *DNMT1* may alter the expression of genes in the GCs to affect follicular growth [18].

Our results showed that the knockdown of *DNMT1* upregulated the level of *SLCO3A1*, promoted the proliferation of GCs, and inhibited the apoptosis of GCs, ultimately fostering follicular growth. These results suggested that *DNMT1*-mediated *SLCO3A1* might serve as a potential therapeutic strategy for the diseases of follicular disorders.

## 4. Materials and Methods

### 4.1. Cells Experiments

The human ovarian granulosa cell line (COV434 cells) utilized in this study was obtained from the cell bank of the Guangdong Provincial Key Laboratory of Agricultural Animal Genome and Molecular Breeding (Guangzhou, China). GCs were cultured with Dulbecco’s modified Eagle’s medium (DMEM, Hyclone, Logan, UT, USA) basal medium containing 10% fetal bovine serum (FBS, Hyclone, Logan, UT, USA) and 1% penicillin–streptomycin (Hyclone, Logan, UT, USA), and they were incubated at 37 °C and 5% CO_2_. When the confluence of GCs reached 70–80%, the transient transfections of plasmids and oligonucleotides were performed using Lipofectamine ™ 3000 reagent (Invitrogen, Waltham, MA, USA). The plasmids included pcDNA3.1 (named OE-NC) and an overexpression plasmid of *SLCO3A1* with pcDNA3.1 as the vector backbone (named OE-SLCO3A1). Oligonucleotides were sourced from Guangzhou Ruibo (Guangzhou, China), and the sequences are shown in Table 1. The RT-qPCR, WB, EdU and flow cytometry assays were performed at 48 h after cell transfection.

### 4.2. Animal Experiments

The vivo experiments were performed on C57BL/6J mice. The 3-week-old female C57BL/6J mice were bought from the Southern Medical University Laboratory Animal Center (Guangzhou, China). We randomly divided the mice into four groups (LV-NC, LV-SLCO3A1, sh-NC, sh-SLCO3A1) with 5 individuals in each group. The lentiviral vectors for the overexpression or knockdown of *SLCO3A1* (LV-SLCO3A1 or sh-SLCO3A1) were synthesized by Guangzhou Dongze (Guangzhou, China). Mice were injected with lentiviral vectors at a dosage of 1 × 10^7^ TU through intraperitoneal injection after 3 days of adaptive feeding. Injections of lentiviral vectors were given once weekly for three weeks. Mice were sacrificed at 42 days of age.

### 4.3. Follicles Culture

Ovaries collected from commercial sows at local slaughterhouses were washed twice with phosphate-buffered saline (PBS) containing 1% penicillin–streptomycin, immersed in PBS, and transported back to the laboratory while maintaining a cold environment. After washing the ovaries with PBS in a sterile laboratory, follicles measuring 3–5 mm in diameter were removed from the ovaries using forceps and scalpels. The follicles were washed twice with medium (DMEM/F12 containing 1% penicillin-streptomycin), then individually placed into twenty-four-well plates with an appropriate volume of medium, and cultured in an incubator at 38.5 °C with 5% CO_2_.

### 4.4. RNA Isolation and Reverse Transcription-Quantitative PCR (RT-qPCR)

Total RNA from tissues and cells was extracted following the instructions provided in the RNAfast200 Total RNA Extraction Kit manual (Feijie, Shanghai, China). The purity and integrity of total RNA were detected using UV spectrophotometer (Thermo Fisher Scientific, Waltham, MA, USA). Afterwards, high-quality RNA was used for cDNA reverse transcription. The reaction system comprised 2 μL of 5× PrimeScript RT premix (TaKaRa, Tokyo, Japan) and ≤500 ng of total RNA, which was adjusted to a final volume of 10 μL with RNase-free H_2_O. Hieff ^®^ qPCR SYBR Green Master Mix (2×) (YEASEN, Shanghai, China) and a CFX96 Touch Real-Time PCR system (Bio-Rad, Berkeley, CA, USA) were used to quantify the relative levels of mRNAs. *GAPDH* was selected as endogenous control, and the 2^−ΔΔct^ method was applied for the analysis of expression level. All primers for RT-qPCR are listed in Table 2, Table 3 and Table 4. The reactions were performed in a total volume of 20 μL per sample, which included 10 μL of SYBR Green Master Mix, 0.6 μL of forward/reverse primer, 1 μL of diluted cDNA template and 7.8 μL of RNA-free water. The cycling conditions were as follows: a holding step at 95 °C for 10 min followed by 40 cycles of 15 s at 95 °C and 1 min at 60 °C.

### 4.5. Western Blot Analysis

Cells and tissues were lysed using RIPA (Bestbio, Shanghai, China) containing 1% Protease Inhibitor (Biosharp, Beijing, China) to obtain protein samples. The total protein concentration was determined using the BCA Protein Assay Kit (Biosharp, Beijing, China). Protein separation was performed by electrophoresis using Future PAGE^TM^ protein precast gels. The separated proteins were transferred to a polyvinylidene fluoride (PVDF) membrane using the eBlot™ L1 membrane converter (GenScript, Nanjing, China). The PVDF membranes were blocked with 5% skim milk powder for 2 h, which was followed by incubation in primary antibody diluent for 12 h. The primary antibodies used were anti-SLCO3A1 (A14276, ABclonal, 1:1000), anti-DNMT1 (DF7376, Affinity, 1:1000), anti-DNMT3A (DF7226, Affinity, 1:1000), anti-DNMT3B (AF5493, Affinity, 1:1000), anti-MCL1 (38113, Signalway, 1:1000), anti-PCNA (60097-1-Ig, Proteintech, 1:10,000), anti-STAR (bs-20387R, Bioss, 1:1000), anti-FSHR (22665-1-AP, Proteintech, 1:1000), anti-CCNE1 (AF4713, Affinity, 1:1000), anti-CDK2 (AF6237, Affinity, 1:1000), anti-CCND1 (AF0931, Affinity, 1:1000), anti-CASP3 (AF6311, Affinity, 1:1000), anti-CASP8 (AF6442, Affinity, 1:1000), anti-CASP9 (AF6348, Affinity, 1:1000), anti-β-Tubulin (10068-1-Ap, Proteintech, 1:1000), anti-GAPDH (YN5585, Immunowau, 1:5000), anti-P65 (10745-1-AP, Proteintech, 1:1000), anti-CDK4 (DF6102, Affinity, 1:1000), anti-CCNB2 (bs-6656R, Bioss, 1:1000), and anti-CDK1 (A0220, Abclonal, 1:1000). The horseradish peroxidase-conjugated goat anti-rabbit immunoglobulin G (IgG) (ab205718, abcam, 1:5000) and goat anti-mouse IgG (L3032-1, Signalway, 1:20,000) were used as the secondary antibodies. The PVDF membranes were incubated in the secondary antibody dilution for 1.5–2 h at room temperature. ECL development solution was added dropwise to the PVDF membrane, and on-line development was performed using a fully automated chemiluminescence image analysis system.

### 4.6. Proliferation Assay

The cell proliferation analysis was conducted using the Cell-Light™ EdU Apollo 567 In Vitro Kit (RiboBio, Guangzhou China). Cells were incubated with 50 μM EdU medium for 2 h, after which the medium was discarded. The cells were then washed twice with PBS for 5 min each time. Following this, cell fixative was added to each well, and the cells were incubated at room temperature for 15–30 min before being washed with PBS for 5 min. Osmolyte was added to each well, and permeabilization was performed for 8 min. Subsequently, the cells were washed with PBS for 5 min. Next, 1 × Apollo staining reaction solution was added to each well, and the cells were incubated for 30 min at room temperature with external foil covering. The cells were then washed with PBS for 5 min, and another round of permeabilization was conducted. DAPI reaction solution was added to each well, which was followed by a 30-min incubation at room temperature with foil covering. Finally, three randomly selected fields of view in each well were observed under a Nikon ECLIPSE Ti2 fluorescence microscope (Nikon, Tokyo, Japan), and images were captured.

### 4.7. Flow Cytometry Assay

The apoptosis rate was detected using an Annexin V-FITC/PI Apoptosis Detection Kit (BD, USA). The cells were collected and washed twice with PBS (Biosharp, Beijing, China), which was followed by gentle suspension in 500 μL of 1× Annexin V Buffer. Subsequently, 5 μL of Annexin V-FITC and 5 μL of propidium iodide staining solution was added to the cells, which were then incubated at 24 °C for 15 min while protected from light. The cell cycle distribution of granulosa cells (GCs) was determined using a cell cycle assay kit (KeyGEN, Nanjing, China). After collection and two washes with PBS, 500 μL of PI/RNase staining buffer was added to each tube of cell samples. The cells were gently resuspended and incubated for 15 min at 37 °C in the absence of light. Finally, the apoptosis rate and cell cycle distribution were analyzed by flow cytometry (BD, Franklin Lakes, NJ, USA) and Flowjo software (version 7.6).

### 4.8. HE Staining

The number of follicles and corpus luteum were detected by HE staining. The largest cross-section cut along the suspensory ligament of the ovary was stained with hematoxylin–eosin, and images were observed and acquired under a Nikon ECLIPSE Ti2 microscope (Nikon, Tokyo, Japan).

### 4.9. TUNEL Assay

The TUNEL method was used to detect apoptosis in mouse ovarian GCs. The sections were deparaffinized using xylene and dehydrated with anhydrous ethanol, which was followed by repair using DNase-free proteinase K. Subsequently, the sections were incubated with 50 μL of the TUNEL assay solution for 60 min at 37 °C while protected from light. After incubation, the sections were washed three times with PBS and then sealed using an anti-fluorescence quenching sealer before observation under a Nikon ECLIPSE Ti2 fluorescence microscope (Nikon, Tokyo, Japan).

### 4.10. Enzyme-Linked Immunosorbent Assay

The Mouse E2 ELISA kit (JM-02849 M2) from Jingmai Biotechnology Co., Ltd. (Jiangsu, China) was employed to determine the concentration of E2 in mouse serum. Specific operational instructions were followed as per the kit manual. The relevant reagents were equilibrated at room temperature for 20 min. Fifty microliters of different standard concentrations was added to the standard wells in the ELISA plate, while 50 μL of 5-fold diluted samples to be tested was added to the sample wells. Subsequently, 100 μL of horseradish peroxidase (HRP)-labeled detection antibody was added to each well, and the reaction wells were sealed with a membrane and incubated at 37 °C for 1 h. After discarding the liquid, each well was filled with washing solution and left to stand for 1 min with the washing step repeated 5 times. Chromogen solution A (50 μL) and chromogen solution B (50 μL) were added to each well, which was followed by incubation at 37 °C away from light for 15 min. Finally, 50 μL of stop solution was added to each well, and the optical density (OD) was read at 450 nm using a microtiter plate reader within 15 min.

### 4.11. Chromatin Accessibility Assay

The chromatin accessibility assay in this study was conducted using the EpiQuik™ Chromatin Accessibility Assay Kit (EPIGENTEK, Farmingdale, NY, USA) following the manufacturer’s instructions. Granulosa cells (GCs) were collected and suspended in 1 × lysis buffer. The cell suspension was divided into sample and No-Nse control groups. Both groups were incubated on ice, which was followed by vortexing and centrifugation to remove the supernatant. The chromatin was washed with 1 mL 1 × wash buffer at 4 °C and centrifuged to discard the supernatant. Subsequently, NDB and Nse were added to the sample group, while the No-Nse control group was treated with NDB only. The Nse reaction mixture was added, which was followed by incubation of the chromatin pellet under specified conditions. DNA was eluted from the binding columns using elution solution (ES) and centrifugation. We designed three primer pairs in the promoter region of *SLOC3A1* (region-1, 282 bp, +971/+1252 bp, region-2, 125 bp, +1433/+1557 bp, and region-3, 209 bp, +1623/+1831 bp, transcription start site = +2000, Table 5). Hieff ^®^ qPCR SYBR Green Master Mix and a CFX96 Touch Real-Time PCR system were used to measure the amplification efficiency. The reaction volume was consistent with that of the RT-qPCR reaction. Amplification programs were performed using a two-step method: pre-denaturation at 95 °C for 10 min, which was followed by 40 cycles including denaturation at 95 °C for 15 s and annealing/extension at 60 °C for 1 min. The fold enrichment (FE) in the *SLCO3A1* promoter region was calculated using the formula *FE* = 2*^(Nse CT − no-Nse CT)^* × 100%.

### 4.12. Statistical Analysis

All statistical analyses were performed with GraphPad Prism software (version 9.0, Boston, MA, USA). The data were expressed as the mean ± SD from at least three independent experiments, and an independent samples *t*-test was used for numerical data. In all cases, * *p* < 0.05, ** *p* < 0.01, *** *p* < 0.001, ^ns^ *p* > 0.05.

## 5. Conclusions

Taken together, we found that the knockdown of *DNMT1* upregulated the level of *SLCO3A1*, leading to a promoted proliferation of GCs, increased E2 secretion, and inhibited apoptosis of GCs, ultimately fostering follicular growth. This suggested that targeting *SLCO3A1* could be a potential strategy for treating female reproductive diseases.

## Figures and Tables

**Table 1:** List of oligonucleotides used in this study.

Sequence Number	Target Sequence (5′→3′)
*SLCO3A1*-siRNA # 1	GCCCTGAACTCAAGTCTTA
*SLCO3A1*-siRNA # 2	CCCACCAGTACAAGTACGA
*SLCO3A1*-siRNA # 3	ACCTGCTCTCAAACCCTGT
siRNA-DNMT1	GGAAGAAGAGUUACUAUAA
siRNA-DNMT3A	GCCUCAGAGCUAUUACCCA
siRNA-DNMT3B	GAAGAUCAAGCUCGCGACU

**Table 2:** Primers used for RT-qPCR in GCs.

Gene Name	Primer Sequences (5′ to 3′)	Size (bp)	Accession Number
*SLCO3A1*	F: GTGGGGTGGCTTTCTGCTCT	145	NM_013272.4
	R: GGGTCTCTCGTATTCTCTTTCGG		
*PCNA*	F: CAAGTAATGTCGATAAAGAGGAGG	126	NM_182649.2
	R: GTGTCACCGTTGAAGAGAGTGG		
*BCL-2*	F: ATCGCCCTGTGGATGACTGAGT	127	NM_000633.3
	R: GCCAGGAGAAATCAAACAGAGGC		
*IKBA*	F: TCCACTCCATCCTGAAGGCTAC	101	NM_020529.3
	R: CAAGGACACCAAAAGCTCCACG		
*STAR*	F: TACGTGGCTACTCAGCATCGAC	142	NM_000349.3
	R: TCAACACCTGGCTTCAGAGGCA		
*MCL1*	F: CCAAGAAAGCTGCATCGAACCAT	151	NM_001197320.2
	R: CAGCACATTCCTGATGCCACCT		
*ESR1*	F: GCTTACTGACCAACCTGGCAGA	129	XM_054354493.1
	R: GGATCTCTAGCCAGGCACATTC		
*ESR2*	F: ATGGAGTCTGGTCGTGTGAAGG	148	NM_001437.3
	R: TAACACTTCCGAAGTCGGCAGG		
*FSHR*	F: GGTTTGTCCTCACCAAGCTTCG	126	NM_000145.4
	R: GGTTGGAGAACACATCTGCCTC		
*CYP1A1*	F: GATTGAGCACTGTCAGGAGAAGC	138	NM_000499.5
	R: ATGAGGCTCCAGGAGATAGCAG		
*CYP17A1*	F: GCACACCAACTATCAGTGACCG	147	NM_000102.4
	R: CCTTGTCCACAGCAAACTCACC		
*CYP19A1*	F: GACGCAGGATTTCCACAGAAGAG	145	NM_001347252.2
	R: ATGGTGTCAGGAGCTGCGATCA		
*HSD17B1*	F: TTCCTGCCAGACATGAAGAGGC	143	NM_001330219.3
	R: AGAACCGCCAGACTCTCGCATA		
*MYC*	F: CCTGGTGCTCCATGAGGAGAC	128	NM_002467.6
	R: CAGACTCTGACCTTTTGCCAGG		
*PAK1*	F: GTGAAGGCTGTGTCTGAGACTC	149	NM_001376274.1
	R: GGAAGTGGTTCAATCACAGACCG		
*CDKN1B*	F: ATAAGGAAGCGACCTGCAACCG	119	NM_004064.5
	R: TTCTTGGGCGTCTGCTCCACAG		
*CCNB2*	F: CAACCAGAGCAGCACAAGTAGC	136	NM_004701.4
	R: GGAGCCAACTTTTCCATCTGTAC		
*CCND1*	F: TCTACACCGACAACTCCATCCG	133	NM_053056.3
	R: TCTGGCATTTTGGAGAGGAAGTG		
*CCNE1*	F: TGTGTCCTGGATGTTGACTGCC	123	NM_001322262.2
	R: CTCTATGTCGCACCACTGATACC		
*CCNE2*	F: CTTACGTCACTGATGGTGCTTGC	126	NM_057749.3
	R: CTTGGAGAAAGAGATTTAGCCAGG		
*CCNH*	F: CGATGTCATTCTGCTGAGCTTGC	128	NM_001199189.2
	R: TCTACCAGGTCGTCATCAGTCC		
*CDK1*	F: GGAAACCAGGAAGCCTAGCATC	124	NM_001320918.1
	R: GGATGATTCAGTGCCATTTTGCC		
*CDK2*	F: ATGGATGCCTCTGCTCTCACTG	97	NM_052827.4
	R: CCCGATGAGAATGGCAGAAAGC		
*CDK4*	F: CCATCAGCACAGTTCGTGAGGT	103	NM_000075.4
	R: TCAGTTCGGGATGTGGCACAGA		
*P53*	F: CCTCAGCATCTTATCCGAGTGG	128	NM_001407269.1
	R: TGGATGGTGGTACAGTCAGAGC		
*BAD*	F: CCAACCTCTGGGCAGCACAGC	126	NM_032989.3
	R: TTTGCCGCATCTGCGTTGCTGT		
*BID*	F: TGGGACACTGTGAACCAGGAGT	125	NM_197966.3
	R: GAGGAAGCCAAACACCAGTAGG		
*CASP3*	F: GGAAGCGAATCAATGGACTCTGG	146	NM_001354783.2
	R: GCATCGACATCTGTACCAGACC		
*CASP7*	F: CGGAACAGACAAAGATGCCGAG	143	NM_033338.6
	R: AGGCGGCATTTGTATGGTCCTC		
*CASP8*	F: GCTGACTTTCTGCTGGGGAT	112	NM_033355.4
	R: GACATCGCTCTCTCAGGCTC		
*CASP9*	F: GTTTGAGGACCTTCGACCAGCT	129	NM_001278054.2
	R: CAACGTACCAGGAGCCACTCTT		
*GAPDH*	F: GTCTCCTCTGACTTCAACAGCG	131	NM_002046.7
	R: ACCACCCTGTTGCTGTAGCCAA		

**Table 3:** Primers used for RT-qPCR in pigs.

Gene Name	Primer Sequences (5′ to 3′)	Size (bp)	Accession Number
*SLCO3A1*	F: GTGGGGTGGCTTTCTGCTCT	145	XM_021099372.1
	R: GGGTCTCTCTATTCTCTTTCGG		
*CCND1*	F: TTCATTTCCAACCCGCCCTC	185	XM_021082686.1
	R: TCCAGAAGGGCTTCGATCTG		
*CDK1*	F: AAGTGTGGCCAGAAGTGGAG	157	XM_005671016.3
	R: CCAGAAATTCGCTTGGCAGG		
*CDK2*	F: TTTGCTGAGATGGTGACCCG	254	NM_001285465.1
	R: GCTGAAATCCGCTTGTTGGG		
*CDK4*	F: TTGTCCGGCTGATGGATGTC	255	NM_001123097.1
	R: GCTTGACTGTCCCACCACTT		
*CCNE1*	F: ACTGATGTCTCTGTTCGCTCC	175	XM_005653265.2
	R: TGTCAGGTGTGGGAATGAAGG		
*BCL-2*	F: GAGTTCGGTGGGGTCATGTG	152	XM_021099593.1
	R: TACAGCTCCACAAAGGCATCC		
*MCL1*	F: GGAAGGCGTTAGAGACCCTG	178	NM_001348806.1
	R: GTCACAATCCTGCCCCAGTT		
*STAR*	F: AGACTTTGTGAGTGTGCGCT	254	NM_213755.2
	R: AGTCCACCTGGGTCTGTGAT		
*P65*	F: CATGCGCTTCCGCTACAAG	284	NM_001114281.1
	R: GGTCCCGCTTCTTTACACAC		
*PCNA*	F: AAGAGGAGGAAGCAGTTACCA	205	NM_001291925.1
	R: TCATCTTCGATCTTGGGAGCC		
*GAPDH*	F: GGACTCATGACCACGGTCCAT	220	XM_021091114.1
	R: TCAGATCCACAACCGACACGT		

**Table 4:** Primers used for RT-qPCR in mice.

Gene Name	Primer Sequences (5′ to 3′)	Size (bp)	Accession Number
*SLCO3A1*	F: CTGCTCCTTCGTTTGTTGGG	279	XM_021167570.2
	R: AGAGGCAAAGAACTCACTGGT		
*BCL-2*	F: CCTGTGGATGACTGAGTACCTG	123	XM_029538378.1
	R: AGCCAGGAGAAATCAAACAGAGG		
*MCL1*	F: AGCTTCATCGAACCATTAGCAGAA	125	NM_008562.3
	R: CCTTCTAGGTCCTGTACGTGGA		
*PCNA*	F: CAAGTGGAGAGCTTGGCAATGG	112	NM_011045.2
	R: GCAAACGTTAGGTGAACAGGCTC		
*SP1*	F: CTCCAGACCATTAACCTCAGTGC	133	XM_029548031.1
	R: CACCACCAGATCCATGAAGACC		
*STAR*	F: GTGCTTCATCCACTGGCTGGAA	113	NM_011485.5
	R: GTCTGCGATAGGACCTGGTTGA		
*CCNB2*	F: GCACTACCATCCTTCTCAGGTG	137	NM_007630.2
	R: TGTGCTGCATGACTTCCAGGAC		
*CCND1*	F: GCAGAAGGAGATTGTGCCATCC	123	NM_001379248.1
	R: AGGAAGCGGTCCAGGTAGTTCA		
*CCNE1*	F: AAGCCCTCTGACCATTGTGTCC	155	XM_021201435.2
	R: CTAAGCAGCCAACATCCAGGAC		
*CDK1*	F: CATGGACCTCAAGAAGTACCTGG	136	NM_007659.4
	R: CAAGTCTCTGTGAAGAACTCGCC		
*CDK2*	F: TCATGGATGCCTCTGCTCTCAC	106	NM_016756.4
	R: TGAAGGACACGGTGAGAATGGC		
*CDK4*	F: CATACCTGGACAAAGCACCTCC	135	XM_029482281.1
	R: GAATGTTCTCTGGCTTCAGGTCC		
*GAPDH*	F: GGACTCATGACCACGGTCCAT	220	NM_008084.4
	R: TCAGATCCACAACCGACACGT		

**Table 5:** Primers used for the chromatin accessibility of the *SLCO3A1* promoter region.

Name	Primer Sequences (5′ to 3′)	Size (bp)
region-1	F: GGGGAGTAATTAGGAGCGGC	282
	R: ACCGAAAGGACAGAAAGGGG	
region-2	F: TCTCCCTTCCTGGGAACAGC	125
	R: GAGACTGGCGGCTCTGC	
region-3	F: CACCCCCCTTACTCCGCAA	209
	R: GCTCTCACCCCCTCAGCCC	

## Data Availability

Data contained within the article.
